# Development and validation of a machine learning–based early warning model for carbapenem-resistant *Klebsiella pneumoniae* bloodstream infections using non-carbapenem susceptibility profiles

**DOI:** 10.3389/fmicb.2026.1807076

**Published:** 2026-04-01

**Authors:** Jiangqin Song, Yujiao Bai, Siyu He, Jinru Ji, Yunbo Chen, Yonghong Xiao

**Affiliations:** 1 Department of Medical Laboratory, The First People’s Hospital of Tianmen City (Tianmen Hospital Affiliated to Wuhan University of Science and Technology), Tianmen, Hubei, China; 2 Hubei Province Key Laboratory of Occupational Hazard Identification and Control, Wuhan University of Science and Technology, Wuhan, Hubei, China; 3 State Key Laboratory for Diagnosis and Treatment of Infectious Diseases, Collaborative Innovation Center for Diagnosis and Treatment of Infectious Diseases, The First Affiliated Hospital, School of Medicine, Zhejiang University, Hangzhou, Zhejiang, China

**Keywords:** bloodstream infection, CRKP, early warning model, machine learning, XGBoost

## Abstract

Carbapenem-resistant *Klebsiella pneumoniae* (CRKP) is a major cause of bloodstream infections with limited therapeutic options. Definitive carbapenem susceptibility results are often obtained late in the laboratory workflow, highlighting the need for early warning tools to support timely risk stratification. We analyzed multicenter surveillance data from the Bloodstream Infection Resistance Surveillance Consortium, including 13,072 *K. pneumoniae* bloodstream isolates collected from 60 hospitals in China between 2014 and 2023. Non-carbapenem antimicrobial susceptibility interpretations were used as model inputs, while carbapenem results were excluded. Data were split chronologically into training (2014–2021), validation (2022), and test (2023) sets. Logistic regression, XGBoost, and CatBoost models were developed and evaluated using discrimination, calibration, decision curve analysis (DCA), and SHAP-based interpretability. XGBoost demonstrated the best overall performance, achieving higher discrimination with a ROC-AUC of 0.993 and a PR-AUC of 0.973 on the test set, along with superior calibration as reflected by the lowest Brier score (0.018). At a sensitivity-targeted threshold (~0.95), XGBoost maintained high sensitivity (0.924), excellent specificity (0.989), and a favorable positive predictive value (0.944), while preserving a high negative predictive value (0.986). SHAP analysis identified key non-carbapenem susceptibility features contributing to CRKP risk prediction. Non-carbapenem susceptibility profiles enable early identification of CRKP bloodstream infections. A machine learning–based early warning model, particularly XGBoost, may support laboratory-based risk stratification and complement conventional susceptibility testing.

## 1 Introduction

Carbapenem-resistant *Klebsiella pneumoniae* (CRKP) is an important cause of bloodstream infections and poses a major challenge for clinical microbiology laboratories due to limited therapeutic options and poor patient outcomes (Yang et al., 2024). CRKP continues to be prioritized in international antimicrobial resistance surveillance programmes.

Conventional antimicrobial susceptibility testing requires bacterial isolation, incubation, and phenotypic interpretation, which typically delays definitive susceptibility results for approximately 24–48 h after blood culture positivity (Blaschke et al., 2012; Satlin et al., 2014). This delay may limit timely optimization of antimicrobial therapy and infection control interventions in patients with bloodstream infections (Ritu and Romney, 2017).

Several previous studies have explored predictive modeling approaches for antimicrobial resistance using clinical, microbiological, or genomic data (Madrigal et al., 2022; Farhat et al., 2023; Liao et al., 2025). For example, machine learning models have been developed to predict carbapenem-resistant Enterobacteriaceae infections using patient-level clinical risk factors or electronic health record data (Yelin et al., 2019). Other studies have applied machine learning algorithms to genomic or phenotypic datasets to predict antimicrobial resistance patterns (Feretzakis et al., 2020, 2021). However, most existing models rely heavily on clinical variables or genomic sequencing data, which may not be routinely available in many clinical microbiology laboratories.

In contrast, the present study focuses exclusively on routinely generated non-carbapenem antimicrobial susceptibility results, which are available during standard laboratory workflows. Furthermore, the model was evaluated using temporal validation, in which models were trained on historical isolates and evaluated on future isolates, thereby better reflecting real-world clinical implementation. To our knowledge, few studies have investigated laboratory-based early warning models for CRKP bloodstream infections using exclusively non-carbapenem susceptibility profiles with temporal validation.

## 2 Materials and methods

The overall study design, including data collection, laboratory processing, model development, and evaluation, is summarized in Supplementary Figure S1.

### 2.1 Data source and study population

This multicenter retrospective study was conducted within the Blood Bacterial Resistant Investigation Collaborative System (BRICS). A total of 13,242 *Klebsiella pneumoniae* bloodstream isolates were collected from 60 tertiary hospitals across 16 provinces in China between 2014 and 2023. After exclusion of duplicate isolates and records with incomplete antimicrobial susceptibility data (Duplicate isolates were defined as isolates obtained from the same patient within a 30-day period, and only the first isolate per patient within this interval was retained.), Isolates with incomplete antimicrobial susceptibility testing results for the non-carbapenem antibiotics used as model features were excluded from analysis, as these records could not be reliably incorporated into the machine learning models. 13,072 non-duplicate isolates were included in the final analysis. The temporal distribution and CRKP prevalence across datasets are summarized in Supplementary Table S1. The annual distribution of antimicrobial susceptibility testing across study cohorts is summarized in Supplementary Table S2.

### 2.2 Bacterial identification and antimicrobial susceptibility testing

All isolates were reconfirmed using MALDI-TOF MS (Vitek MS, bioMérieux, France). Antimicrobial susceptibility testing was performed manually using standardized broth microdilution or agar dilution methods in accordance with Clinical and Laboratory Standards Institute (CLSI) guidelines applicable during the study period (2014–2023). All susceptibility interpretations were based on CLSI breakpoints corresponding to the year of testing. Quality control procedures were conducted using standard reference strains as recommended by CLSI to ensure accuracy and reproducibility of results.

### 2.3 Feature construction and outcome definition

Only non-carbapenem antimicrobial susceptibility interpretations were used as model input features to enable early laboratory-based prediction. Susceptibility results for imipenem, meropenem, and ertapenem were explicitly excluded to prevent information leakage. Susceptibility interpretations were encoded numerically as S = 0, I = 0.5, and R = 1.

The outcome of interest was CRKP, defined phenotypically according to CLSI carbapenem breakpoints, and treated as a binary classification task.

### 2.4 Temporal data splitting strategy

To mimic real-world clinical deployment, data were split chronologically rather than randomly. Isolates from 2014–2021 were used for model training, 2022 for validation, and 2023 for independent testing. This design ensured temporal generalizability and minimized information leakage.

### 2.5 Model development

Three machine learning models were developed and compared: logistic regression (baseline, baseline interpretable comparator), XGBoost (primary model, high performance for structured tabular data), and CatBoost (robustness assessment, robustness to categorical-like inputs and overfitting control). Class imbalance was addressed using class-weighting strategies. Model hyperparameters were optimized on the validation dataset.

#### 2.5.1 Logistic regression

Logistic regression was used as the baseline model due to its interpretability and widespread application in clinical prediction modeling. The model estimates the log-odds of CRKP occurrence as a linear combination of input features, allowing direct assessment of feature coefficients. To reduce potential overfitting and improve numerical stability, features were standardized and class weights were incorporated to account for imbalance between CRKP and non-CRKP isolates. Maximum likelihood estimation was used for parameter optimization.

#### 2.5.2 XGBoost

XGBoost (Extreme Gradient Boosting) is an ensemble tree-based algorithm that builds additive decision trees in a stage-wise manner using gradient boosting. At each iteration, the model fits a new tree to the negative gradient of the loss function, thereby minimizing classification error through sequential residual correction. XGBoost incorporates second-order gradient information, regularization terms, and shrinkage to enhance robustness and prevent overfitting.

Given the structured tabular nature of antimicrobial susceptibility data, tree-based boosting is well-suited for capturing nonlinear relationships and high-order feature interactions. Class imbalance was handled using the scale_pos_weight parameter, defined as the ratio of negative to positive samples in the training set. Hyperparameters—including number of trees, learning rate, and maximum tree depth—were optimized using validation-set performance.

#### 2.5.3 CatBoost

CatBoost is also a gradient boosting decision tree algorithm but introduces ordered boosting and specialized handling of categorical features to reduce prediction shift and target leakage. Although susceptibility interpretations were numerically encoded, CatBoost’s ordered boosting mechanism improves generalization under structured clinical data settings. Similar to XGBoost, class weights were incorporated to address imbalance. Model parameters, including tree depth and learning rate, were tuned using validation data.

Handling of class imbalance: CRKP prevalence was lower than non-CRKP across cohorts. To mitigate bias toward the majority class, class weighting strategies were applied in all models. In addition, threshold selection was guided by a predefined sensitivity target (~0.95) to align with the early-warning objective of minimizing false negatives.

Hyperparameter optimization strategy: hyperparameters were selected based on performance in the temporal validation cohort (2022 isolates). The objective was to maximize discrimination (ROC-AUC) while maintaining high sensitivity. Final model configurations were then evaluated in the independent 2023 test cohort to assess generalizability.

Deep learning models were not explored because the dataset comprised structured laboratory features without high-dimensional representations, and model interpretability was prioritized for clinical applicability.

### 2.6 Model evaluation and clinical utility

Model discrimination was assessed using ROC-AUC and PR-AUC, while calibration was evaluated using calibration curves and Brier scores. For early-warning purposes, classification thresholds were determined on the validation set by fixing sensitivity at approximately 0.95. Clinical utility was evaluated using decision curve analysis (DCA) across a range of threshold probabilities.

Because carbapenem-resistant *K. pneumoniae* isolates represented a minority of the dataset, class imbalance was present in the training data. In the training cohort, CRKP accounted for approximately 20.85% of isolates, see Supplementary Table S1, while the remaining isolates were carbapenem-susceptible.

To mitigate the potential impact of class imbalance on model training, class weighting strategies were applied during model development. Specifically, higher weights were assigned to the minority class (CRKP) during training of the logistic regression and gradient boosting models, allowing the models to place greater emphasis on correctly identifying resistant isolates.

### 2.7 Model interpretability

Model interpretability was assessed for the XGBoost model using SHapley Additive exPlanations (SHAP). Global feature importance and individual-level contributions were analyzed to facilitate clinical interpretability.

### 2.8 Statistical analysis

Antimicrobial susceptibility data were analyzed using WHONET 5.6 software. Other analyses were conducted using Python (version 3.11.9), with major libraries including scikit-learn, xgboost, catboost, and shap.

## 3 Results

### 3.1 Dataset characteristics

A total of 13,072 non-duplicate *Klebsiella pneumoniae* bloodstream isolates were included in the final analysis. According to the predefined temporal splitting strategy, 7,703 isolates collected between 2014 and 2021 were assigned to the training set, 2,340 isolates from 2022 to the validation set, and 3,030 isolates from 2023 to the independent test set (Table S1).

The prevalence of CRKP differed across datasets. The training set showed an overall CRKP proportion of 20.85%, with annual prevalence ranging from 13.54 to 31.20% between 2014 and 2021. In comparison, the CRKP proportion was 13.03% in the validation set and 16.11% in the test set (Supplementary Table S1).

This temporal separation ensured that model training, validation, and testing were conducted on isolates collected in distinct time periods, enabling assessment of temporal generalizability.

### 3.2 ROC, PR, and calibration curves

**Figure 1:**
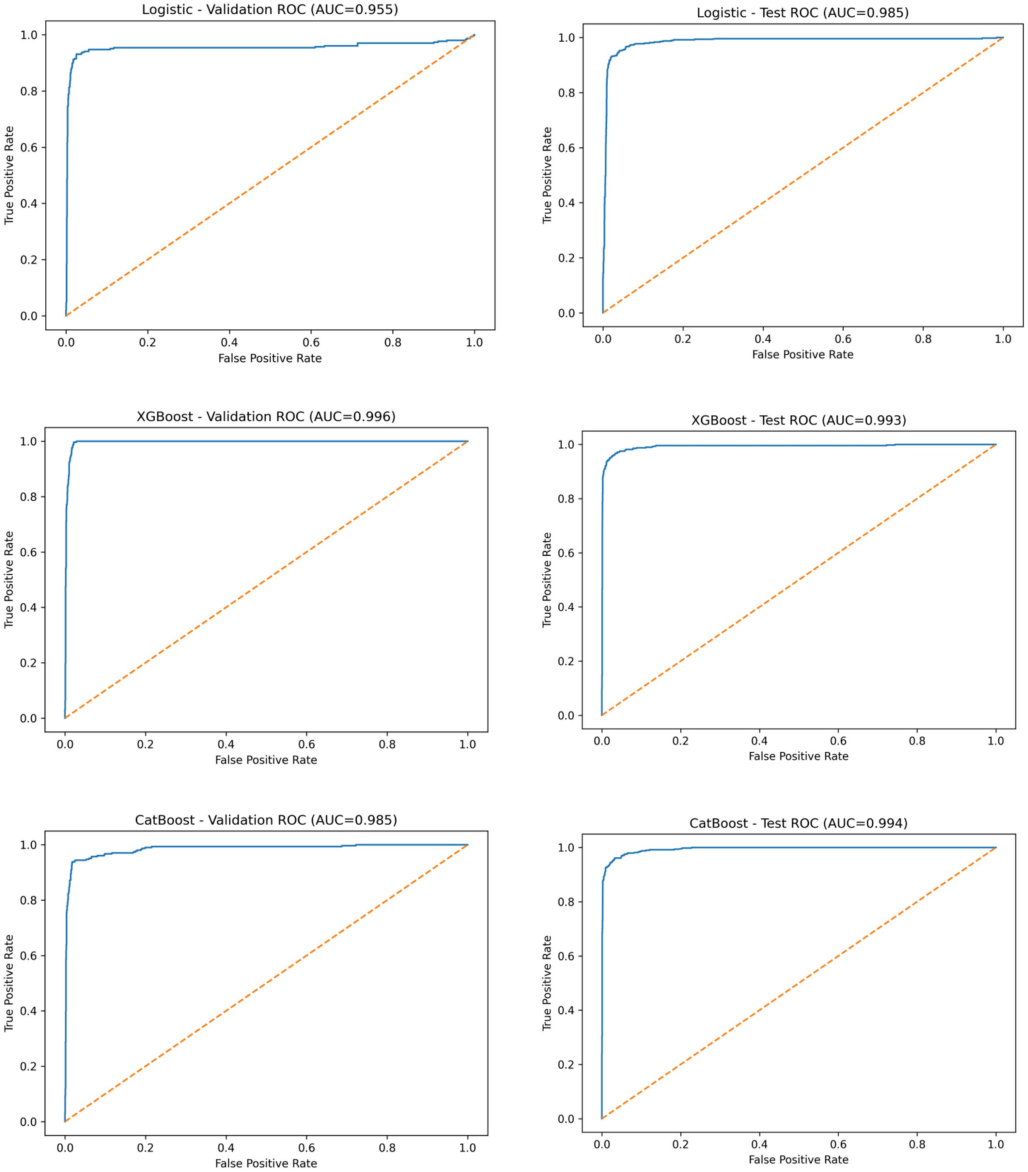
Receiver operating characteristic (ROC) curves of three predictive models for CRKP identification in the validation and test cohorts. Receiver operating characteristic (ROC) curves comparing the discriminative performance of logistic regression, XGBoost, and CatBoost models for predicting CRKP.

Receiver operating characteristic (ROC) curves for the three models in the validation and test cohorts are shown in Figure 1. In both cohorts, XGBoost and CatBoost consistently demonstrated higher ROC curves than Logistic Regression across most false-positive rate ranges.

**Figure 2:**
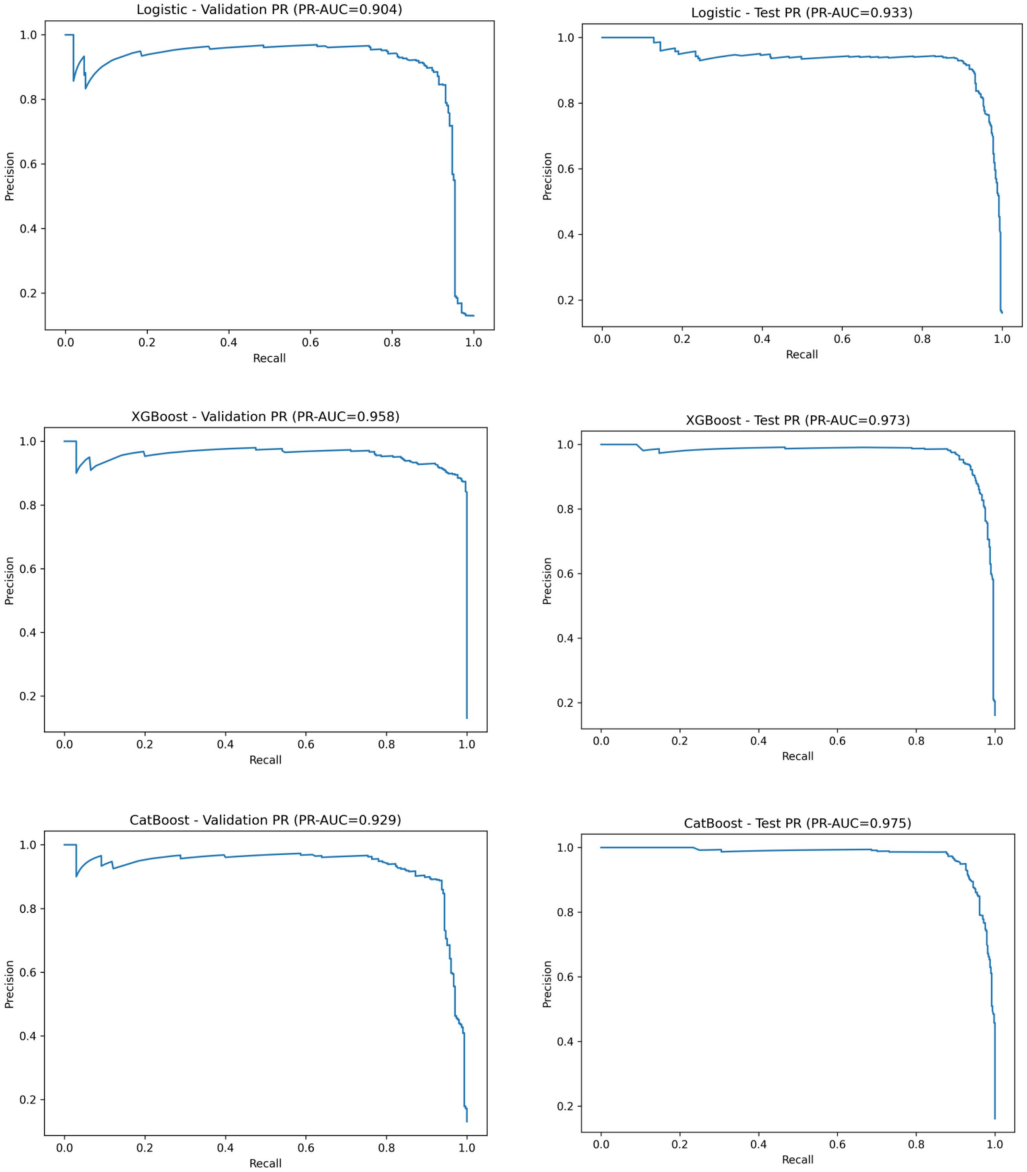
Precision–recall (PR) curves of three machine learning models for CRKP prediction in the validation and test cohorts. Precision–recall (PR) curves comparing the performance of Logistic Regression, XGBoost, and CatBoost models for predicting CRKP using routine non-carbapenem antimicrobial susceptibility test results.

Precision–recall (PR) curves are presented in Figure 2. Under class-imbalanced conditions, XGBoost and CatBoost maintained higher precision across a wide range of recall values compared with Logistic Regression in both the validation and test cohorts.

**Figure 3:**
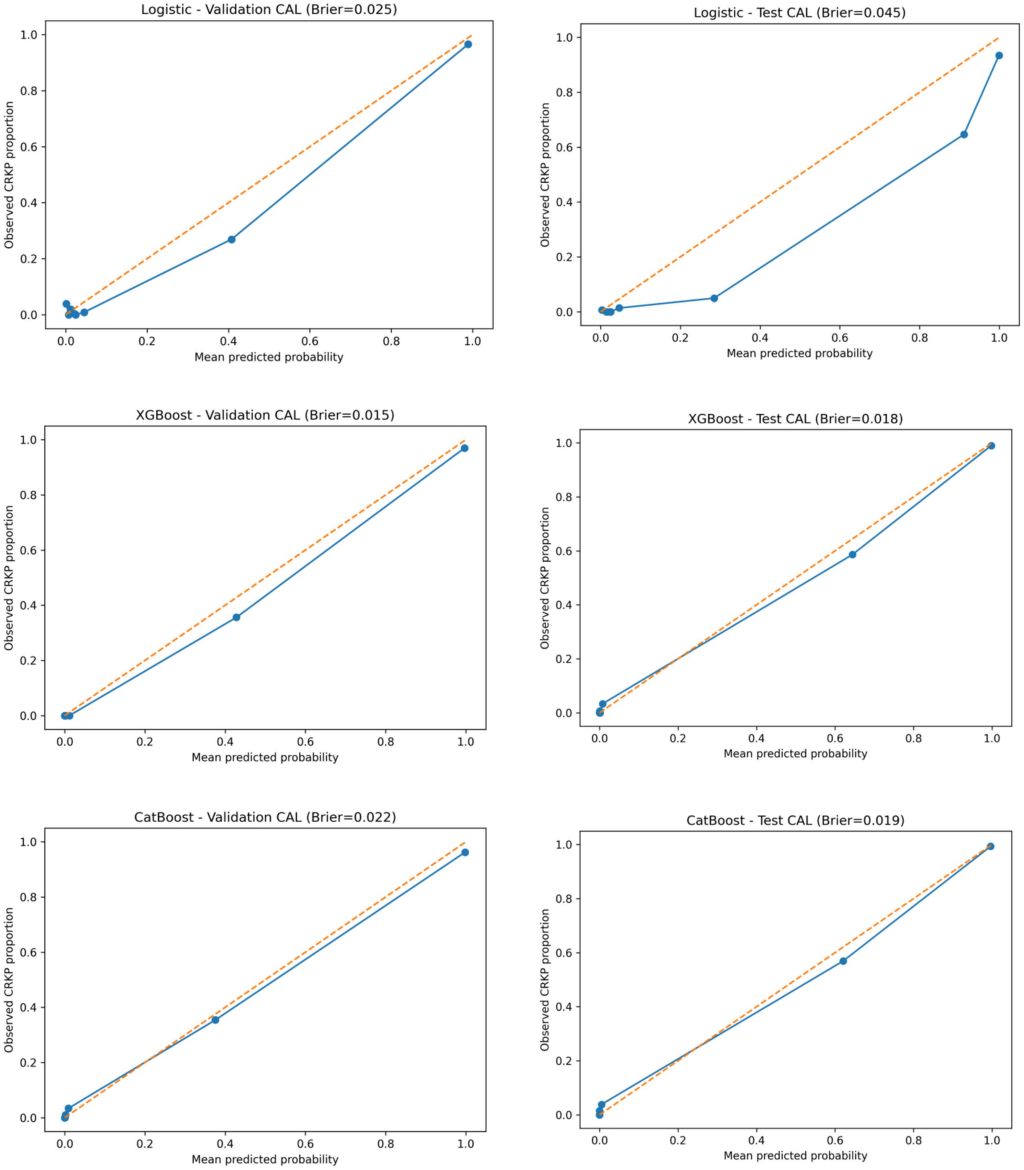
Calibration performance of three predictive models for CRKP identification in the validation and test cohorts. Calibration curves of the logistic regression, XGBoost, and CatBoost models in the validation (2022) and test (2023) cohorts.

Calibration performance is illustrated in Figure 3. In both cohorts, predicted probabilities from XGBoost and CatBoost closely aligned with the observed CRKP proportions across probability bins. In contrast, Logistic Regression showed larger deviations from the ideal calibration line, particularly at higher predicted probability ranges. These observations were consistent with the Brier scores reported in Table 1.

**Table 1:** Performance comparison of predictive models for CRKP identification.

Model	Cohort	ROC-AUC	PR-AUC	Brier score	Sensitivity	Specificity	PPV	NPV
Logistic regression	Validation	0.955	0.904	0.025	0.950*			
	Test	0.985	0.933	0.045	0.992	0.810	0.501	0.998
XGBoost	Validation	0.996	0.958	0.015	0.950*			
	Test	0.993	0.973	0.018	0.924	0.989	0.944	0.986
CatBoost	Validation	0.985	0.929	0.022	0.950*			
	Test	0.994	0.975	0.019	0.961	0.954	0.802	0.992

*Sensitivity was fixed at 0.95 in the validation cohort to determine the operating threshold.

### 3.3 Model discrimination performance

The discriminative performance of the three predictive models—Logistic Regression, XGBoost, and CatBoost—was evaluated in both the validation and independent test cohorts. Overall model performance metrics are summarized in Table 1.

In the validation cohort, XGBoost achieved the highest discrimination, with a ROC-AUC of 0.996 and a PR-AUC of 0.958, followed by CatBoost (ROC-AUC 0.985, PR-AUC 0.929) and Logistic Regression (ROC-AUC 0.955, PR-AUC 0.904).

In the independent test cohort, all models maintained high discriminative performance. CatBoost yielded the highest ROC-AUC (0.994), closely followed by XGBoost (0.993), while Logistic Regression achieved a ROC-AUC of 0.985. Precision–recall analysis showed that XGBoost (PR-AUC 0.973) and CatBoost (PR-AUC 0.975) outperformed Logistic Regression (PR-AUC 0.933) in the test set.

### 3.4 Clinical utility assessed by decision curve analysis

**Figure 4:**
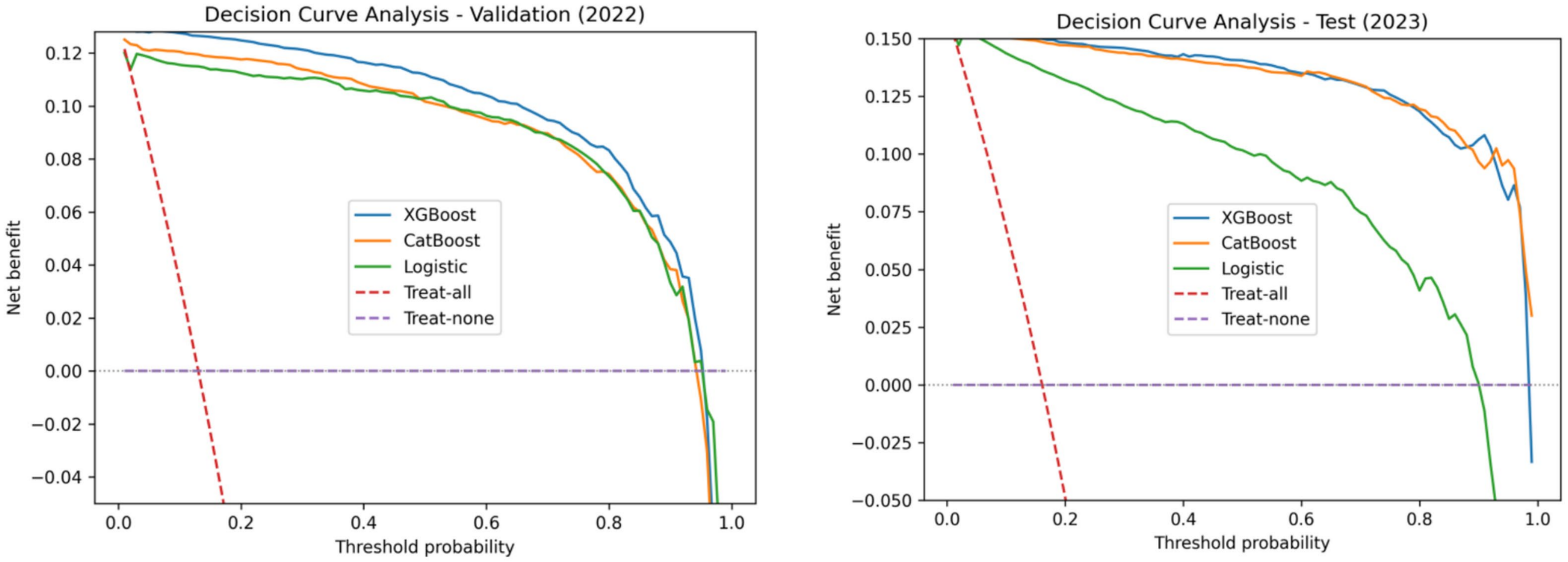
DCA of three machine learning models for predicting carbapenem-resistant *Klebsiella pneumoniae* bloodstream infection. DCA comparing the clinical net benefit of logistic regression, XGBoost, and CatBoost models for predicting CRKP bloodstream infection in the validation cohort (2022) and the independent test cohort (2023). The *x*-axis represents the threshold probability, and the *y*-axis represents the net benefit. The dashed red line indicates the “treat-all” strategy, while the dashed purple line indicates the “treat-none” strategy. Models demonstrating a higher net benefit than both default strategies across a clinically relevant range of threshold probabilities are considered to have potential clinical utility.

Decision curve analysis (DCA) was performed to evaluate the net clinical benefit of the three models across a range of threshold probabilities in both the validation and test cohorts (Figure 4).

In the validation cohort, all three models yielded higher net benefit than the “treat-all” and “treat-none” strategies across a broad range of clinically relevant threshold probabilities. Among the models, XGBoost and CatBoost consistently demonstrated higher net benefit than Logistic Regression, particularly at moderate threshold probabilities.

Similar patterns were observed in the independent test cohort. XGBoost maintained the highest net benefit across the widest threshold probability range, followed closely by CatBoost, while Logistic Regression showed positive net benefit over a narrower interval.

### 3.5 Model interpretability using SHAP

**Figure 5:**
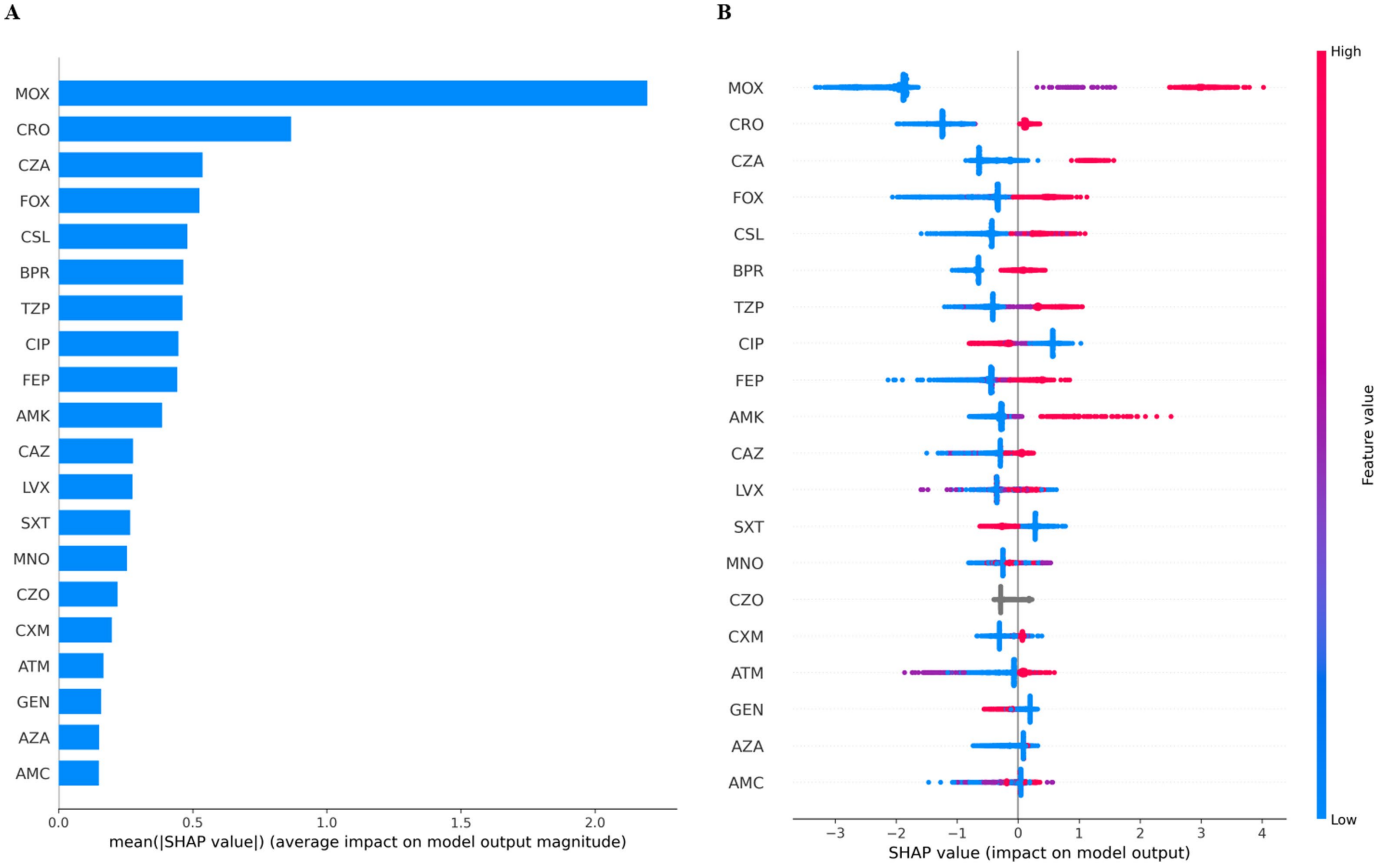
SHAP-based feature importance and contribution patterns of the XGBoost model for predicting CRKP bloodstream infections. SHAP analysis illustrating feature importance and directionality of contributions in the XGBoost model. **(A)** Bar plot showing the mean absolute SHAP values for each antimicrobial susceptibility variable, representing the average contribution magnitude to the model output across all samples. **(B)** SHAP beeswarm plot displaying the distribution of SHAP values for individual samples, where each point represents one isolate. Colors indicate feature values (blue: low, red: high). Positive SHAP values correspond to increased predicted risk of carbapenem-resistant *Klebsiella pneumoniae* (CRKP), whereas negative values indicate decreased predicted risk.

Model interpretability was assessed for the XGBoost model using SHapley Additive exPlanations (SHAP). Global feature importance is shown in Figure 5A, where several non-carbapenem antimicrobial susceptibility variables demonstrated high mean absolute SHAP values, including moxalactam (MOX), ceftriaxone (CRO), ceftazidime-related variables, cefoxitin (FOX), and cefoperazone/sulbactam (CSL).

The SHAP beeswarm plot (Figure 5B) illustrated the distribution and directionality of feature contributions across individual isolates. Higher resistance-associated feature values were predominantly associated with positive SHAP values, corresponding to increased predicted probability of CRKP, whereas susceptible or intermediate profiles were associated with negative SHAP values.

These results demonstrate that the XGBoost model integrates information from multiple non-carbapenem antimicrobial susceptibility profiles to generate CRKP risk predictions.

## 4 Discussion

In this multicenter study, we developed and temporally validated a machine learning–based early warning model for predicting CRKP bloodstream infections using routinely available non-carbapenem antimicrobial susceptibility profiles (Long et al., 2025). By leveraging a large national surveillance dataset and a strict chronological split, the model was evaluated under conditions closely resembling real-world clinical deployment.

Temporal variation in CRKP prevalence was observed across the study period, and the overall CRKP proportion in the BRICS dataset was higher than that reported by national surveillance networks such as China Antimicrobial Resistance Surveillance System(CARSS) and China Antimicrobial Surveillance Network(CHINET), CHINET surveillance data demonstrated that the prevalence of CRKP showed a remarkable increasing trend from 2.9% (imipenem resistance) in 2005 to 25.0% in 2018, and then slightly decreased to 22.6% in 2022 (Qin et al., 2024). This difference is likely attributable to variations in surveillance design rather than laboratory inconsistency. First, BRICS exclusively collects *Klebsiella pneumoniae* isolates causing bloodstream infections, whereas both CARSS and CHINET aggregate isolates from all clinical specimen types, with respiratory tract samples accounting for approximately 40–50% of all isolates in these networks (Wang et al., 2025; Hu et al., 2019). Respiratory isolates are more likely to include colonizing or non-invasive strains (Golden et al., 2019), which may substantially bias resistance estimates and limit their ability to reflect the true resistance burden among invasive pathogens. Correcting such specimen-related bias was one of the primary motivations for establishing the BRICS network, which focuses solely on clinically significant invasive infections (Dai et al., 2021). Second, before 2020, the number of participating centers in the BRICS network was relatively limited and mainly consisted of large tertiary teaching hospitals, which may partly account for the higher observed CRKP prevalence during this period (Wang et al., 2021; Kong et al., 2021). Temporal analyses from BRICS as well as national surveillance networks such as CARSS and CHINET consistently indicate a declining trend in CRKP prevalence in recent years (Hu et al., 2018). Importantly, these factors do not undermine the internal validity or clinical utility of the present predictive modeling.

One of the principal findings of this study is that non-carbapenem susceptibility patterns contain sufficient predictive information to support early identification of CRKP (Lou et al., 2022). Among the three evaluated models, XGBoost consistently demonstrated superior overall performance, with high discrimination, favorable calibration, and the greatest net benefit in decision curve analysis. These results were observed not only in the validation cohort but also in an independent test cohort collected in a subsequent calendar year, supporting the temporal robustness of the model.

The use of a chronological training–validation–testing strategy represents an important methodological strength (Ramirez-Campillo et al., 2023). Unlike random splitting, which may artificially inflate performance by allowing temporal information leakage, the present design ensured that model evaluation was conducted on isolates collected after model development. This approach provides a more conservative and clinically relevant estimate of predictive performance, particularly for applications intended for prospective use (Coley et al., 2021; Coley et al., 2023).

Clinical utility was explicitly assessed using decision curve analysis, which demonstrated that the XGBoost model yielded higher net benefit than treat-all or treat-none strategies across a wide range of threshold probabilities. This finding supports the intended role of the model as an early warning tool rather than a definitive diagnostic test. By prioritizing sensitivity during threshold selection, the model aligns with infection control and antimicrobial stewardship scenarios in which early identification of high-risk cases is clinically desirable.

The precision–recall performance and modest positive predictive values observed in this study should be interpreted in the context of the underlying prevalence of CRKP. In settings where CRKP prevalence is relatively low, even models with excellent discrimination may yield limited positive predictive values (Liao et al., 2023). In early warning applications, maintaining high sensitivity while achieving acceptable specificity and net clinical benefit is often more relevant than maximizing positive predictive value alone (Hung et al., 2025).

Model interpretability was addressed using SHAP analysis (Bhandari et al., 2023; Li et al., 2024), which demonstrated that the XGBoost model incorporated contributions from multiple non-carbapenem antimicrobial susceptibility variables. The distributed pattern of feature importance suggests that predictions were not driven by a single antimicrobial agent but rather by integrated resistance profiles across several drug classes (Fan et al., 2025), enhancing the transparency and credibility of the model’s predictions.

Several limitations should be acknowledged. First, this study was retrospective in nature and relied exclusively on laboratory-based antimicrobial susceptibility data without incorporating clinical variables such as prior antibiotic exposure or patient comorbidities. Second, although the dataset was large and multicenter, external validation in non-BRICS populations was not performed. Finally, changes in antimicrobial prescribing practices and resistance epidemiology over time may influence model performance and warrant periodic model updating.

To clarify the intended application, the proposed model is not designed to replace carbapenem susceptibility testing. Rather, it functions as a laboratory-based early risk stratification tool. While carbapenem and non-carbapenem susceptibility tests are often performed simultaneously, carbapenem results may require confirmatory testing or repeat MIC determination when resistance is suspected or results appear inconsistent. Given the infection-control implications of CRKP, such verification processes can extend reporting time by several hours. In this context, leveraging routinely available non-carbapenem susceptibility profiles may provide preliminary risk information to enhance early awareness and antimicrobial stewardship decision-making.

In conclusion, this study demonstrates that machine learning models based on non-carbapenem susceptibility profiles can provide effective early warning of CRKP bloodstream infections. The XGBoost model showed favorable discrimination, calibration, and clinical utility under temporal validation, supporting its potential role as a laboratory-based decision support tool for early risk stratification.

## 5 Conclusion

In this multicenter study of 13,072 bloodstream *Klebsiella pneumoniae* isolates, we developed and temporally validated a laboratory-based machine learning model for early identification of CRKP using non-carbapenem susceptibility profiles. Among the evaluated algorithms, XGBoost demonstrated superior discrimination, calibration, and clinical utility across independent validation and test cohorts. By leveraging routinely available antimicrobial susceptibility data prior to definitive carbapenem interpretation, the proposed model enables early risk stratification and may support timely infection control and empirical treatment decisions. Future work should focus on prospective validation and integration into real-time laboratory information systems.

## Data Availability

The raw data supporting the conclusions of this article will be made available by the authors, without undue reservation.

## Supplementary material

SUPPLEMENTARY FIGURE S1 Workflow of the study for developing and validating a laboratory-based early warning model for carbapenem-resistant *Klebsiella pneumoniae* (CRKP). Bloodstream infection isolates were collected through the BRICS network, processed by standardized identification and susceptibility testing, temporally split into training, validation, and test sets, and used to develop and evaluate machine learning models based on non-carbapenem susceptibility profiles.
