# Supplementary material for: Development and validation of a machine learning–based early warning model for carbapenem-resistant Klebsiella pneumoniae bloodstream infections using non-carbapenem susceptibility profiles
Source: Front Microbiol. 2026 Apr 1;17:1807076. doi: 10.3389/fmicb.2026.1807076 (PMC13081778; doi:10.3389/fmicb.2026.1807076)
Supplement: Supplementary file 1 [file Table_1.docx]

**Table S1 Temporal distribution and prevalence of CRKP across study cohorts**

| **Cohort** | **Year(s)** | **No. of KPN isolates**  **（Percentage）** | **No. of CRKP isolates** | **CRKP proportion (%)** |
| --- | --- | --- | --- | --- |
| Training set | 2014-2021 | 7703(58.92%) | 1606 | 20.85 |
|  | 2014 | 354 | 68 | 19.21 |
|  | 2015 | 583 | 166 | 28.47 |
|  | 2016 | 752 | 140 | 18.62 |
|  | 2017 | 657 | 205 | 31.20 |
|  | 2018 | 989 | 294 | 29.73 |
|  | 2019 | 1250 | 290 | 23.20 |
|  | 2020 | 1331 | 201 | 15.10 |
|  | 2021 | 1787 | 242 | 13.54 |
| Validation set | 2022 | 2340(17.89%) | 305 | 13.03 |
| Test set | 2023 | 3030(23.17%) | 488 | 16.11 |
| Total |  | 13072 | 2399 | 18.35 |
