# Supplementary material for: Development and validation of a machine learning–based early warning model for carbapenem-resistant Klebsiella pneumoniae bloodstream infections using non-carbapenem susceptibility profiles
Source: Front Microbiol. 2026 Apr 1;17:1807076. doi: 10.3389/fmicb.2026.1807076 (PMC13081778; doi:10.3389/fmicb.2026.1807076)
Supplement: Supplementary file 2 [file Table_2.docx]

**Table S2 Annual distribution of antimicrobial susceptibility testing across study cohorts.**

| **Dataset** | **Year** | **AMC** | **AMK** | **AMX** | **ATM** | **AZA** | **BPR** | **CAZ** | **CIP** | **CRO** | **CSL** | **CXM** | **CZA** | **CZO** | **FEP** | **FOS** | **FOX** | **GEN** | **IMR** | **LVX** | **MNO** | **MOX** | **PIP** | **POL** | **SXT** | **TGC** | **TZP** |
| --- | --- | --- | --- | --- | --- | --- | --- | --- | --- | --- | --- | --- | --- | --- | --- | --- | --- | --- | --- | --- | --- | --- | --- | --- | --- | --- | --- |
| Train | 2014 | 223 | 353 | 1 | 353 | 0 | 0 | 353 | 353 | 353 | 223 | 223 | 0 | 353 | 354 | 223 | 223 | 353 | 0 | 353 | 0 | 223 | 0 | 223 | 353 | 223 | 223 |
| Train | 2015 | 343 | 582 | 7 | 582 | 0 | 0 | 583 | 582 | 582 | 343 | 343 | 0 | 524 | 582 | 343 | 343 | 582 | 0 | 583 | 0 | 343 | 0 | 343 | 583 | 343 | 343 |
| Train | 2016 | 695 | 747 | 2 | 749 | 0 | 0 | 747 | 747 | 749 | 695 | 695 | 0 | 741 | 747 | 695 | 695 | 747 | 0 | 747 | 0 | 695 | 0 | 695 | 749 | 695 | 695 |
| Train | 2017 | 652 | 652 | 0 | 654 | 0 | 0 | 565 | 652 | 653 | 565 | 565 | 0 | 649 | 653 | 565 | 652 | 653 | 0 | 652 | 0 | 565 | 0 | 565 | 653 | 565 | 565 |
| Train | 2018 | 986 | 989 | 0 | 898 | 0 | 0 | 947 | 896 | 989 | 946 | 944 | 0 | 898 | 989 | 855 | 986 | 898 | 0 | 983 | 0 | 855 | 0 | 864 | 989 | 855 | 855 |
| Train | 2019 | 1221 | 1249 | 1 | 1205 | 0 | 0 | 1247 | 1205 | 1221 | 1248 | 1223 | 1177 | 1177 | 1249 | 1177 | 1222 | 1178 | 0 | 1248 | 0 | 1177 | 0 | 1189 | 1248 | 1177 | 1178 |
| Train | 2020 | 1331 | 1331 | 1252 | 1331 | 0 | 0 | 1331 | 1331 | 1331 | 1331 | 1331 | 1331 | 1331 | 1331 | 1331 | 1331 | 1331 | 0 | 1331 | 1252 | 1331 | 1252 | 1331 | 1331 | 1331 | 1331 |
| Train | 2021 | 1787 | 1787 | 1787 | 1787 | 1427 | 1427 | 1787 | 1787 | 1787 | 1786 | 1787 | 1787 | 1787 | 1787 | 1787 | 1787 | 1787 | 0 | 1787 | 1787 | 1787 | 1787 | 1787 | 1787 | 1787 | 1787 |
| Validation | 2022 | 2322 | 2323 | 2339 | 2315 | 2339 | 2321 | 2321 | 2324 | 2326 | 2317 | 2340 | 2338 | 2340 | 2319 | 2335 | 2324 | 2323 | 2340 | 2324 | 2338 | 2322 | 2340 | 2331 | 2338 | 2340 | 2320 |
| Test | 2023 | 3030 | 3030 | 0 | 3030 | 3030 | 3030 | 3030 | 3030 | 3030 | 3030 | 3030 | 3030 | 0 | 3030 | 3028 | 3030 | 3030 | 3030 | 3030 | 3030 | 3030 | 0 | 3030 | 3030 | 3030 | 3030 |

Note：The training cohort included isolates collected between 2014 and 2021, the validation cohort comprised isolates from 2022, and the test cohort included isolates from 2023. Values represent annual numbers of antimicrobial susceptibility tests performed for each antibiotic. Abbreviations: AMC, amoxicillin–clavulanic acid; AMK, amikacin; AMX, amoxicillin; ATM, aztreonam; AZA, azithromycin; BPR, cefoperazone–sulbactam; CAZ, ceftazidime; CIP, ciprofloxacin; CRO, ceftriaxone; CSL, cefoperazone–sulbactam (CLSI code); CXM, cefuroxime; CZA, ceftazidime–avibactam; CZO, cefazolin; FEP, cefepime; FOS, fosfomycin; FOX, cefoxitin; GEN, gentamicin; IMR, imipenem; LVX, levofloxacin; MNO, minocycline; MOX, moxalactam; PIP, piperacillin; POL, polymyxin B; SXT, trimethoprim–sulfamethoxazole; TGC, tigecycline; TZP, piperacillin–tazobactam.
