# Supplementary figures and images for: Development and validation of a machine learning–based early warning model for carbapenem-resistant Klebsiella pneumoniae bloodstream infections using non-carbapenem susceptibility profiles
Source: Front Microbiol. 2026 Apr 1;17:1807076. doi: 10.3389/fmicb.2026.1807076 (PMC13081778; doi:10.3389/fmicb.2026.1807076)

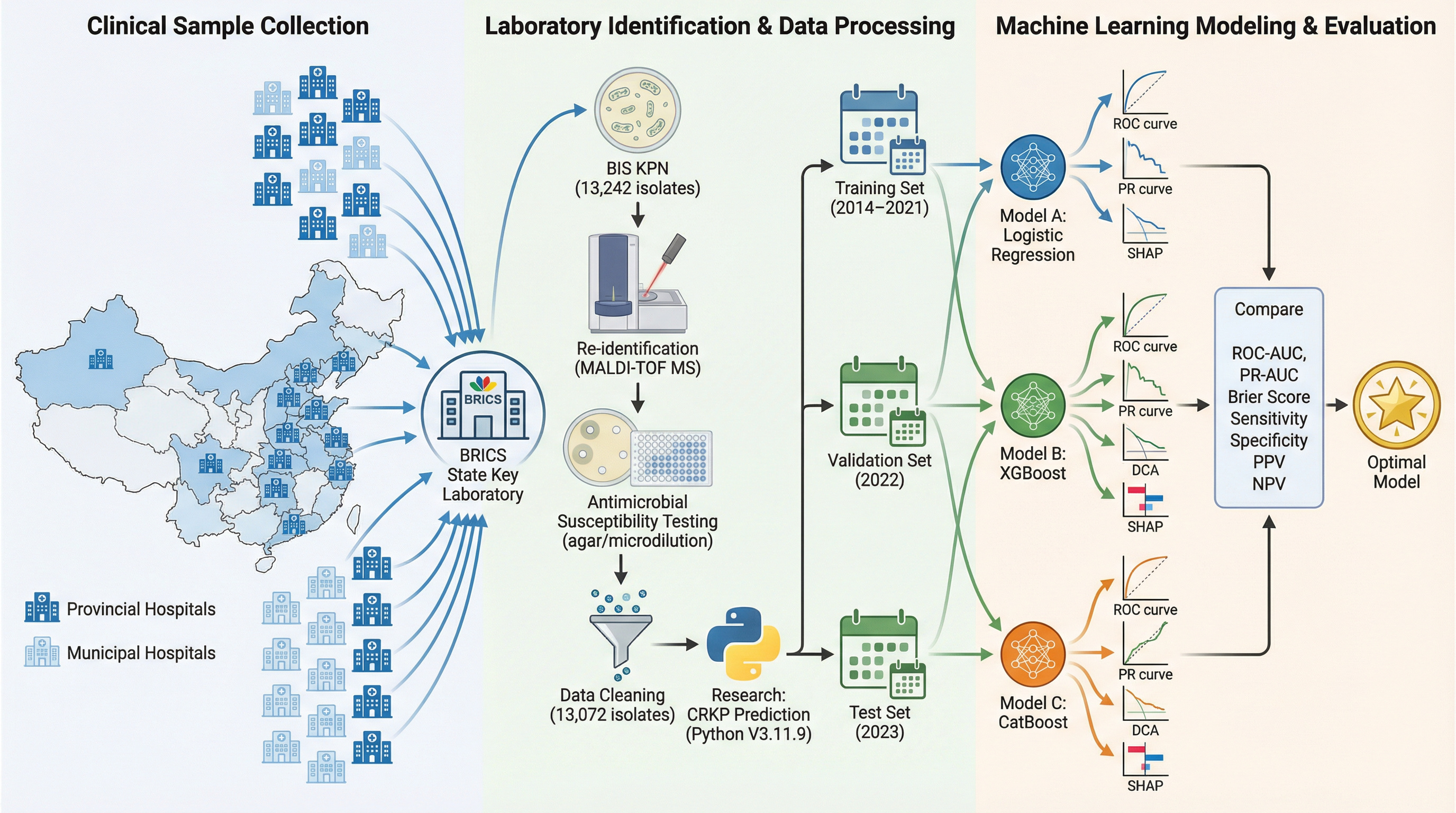

Supplement: SUPPLEMENTARY FIGURE S1 — Workflow of the study for developing and validating a laboratory-based early warning model for carbapenem-resistant Klebsiella pneumoniae (CRKP). Bloodstream infection isolates were collected through the BRICS network, processed by standardized identification and susceptibility testing, temporally split into training, validation, and test sets, and used to develop and evaluate machine learning models based on non-carbapenem susceptibility profiles. [file Image_1.png]
